# Psoriatic Arthritis Involving TMJ: A Review on Pathogenesis and Consideration on Eventual Gender Differences

**DOI:** 10.3390/dj12020031

**Published:** 2024-02-02

**Authors:** Sara Bernardi, Lucia Memè, Chiara Belfioretti, Fabrizio Bambini, Davide Gerardi, Guido Macchiarelli, Serena Bianchi, Stefano Mummolo

**Affiliations:** 1 Department of Life, Health and Environmental Sciences, University of L’Aquila, 67100 L’Aquila, Italy; sara.bernardi@univaq.it (S.B.); davide.gerardi@studenti.unich.it (D.G.); guido.macchiarelli@univaq.it (G.M.); serena.bianchi@univaq.it (S.B.); stefano.mummolo@univaq.it (S.M.); 2 Department of Clinical Sciences and Stomatology, Polytechnic University of Marche, 60126 Ancona, Italy; chiara.belfioretti.an@gmail.com (C.B.); f.bambini@staff.univpm.it (F.B.); 3 Department of Innovative Technologies in Medicine & Dentistry, Dental School, ‘G. D’Annunzio’ University of Chieti-Pescara, 66100 Chieti, Italy

**Keywords:** psoriatic arthritis, TMJ, gender medicine, women’s health

## Abstract

Psoriatic arthritis is defined as chronic inflammatory arthritis associated with psoriasis. The current data regarding gender differences in clinical manifestation and therapeutic outcomes of psoriatic arthritis are limited. Generally, men show a peripheral disease manifestation, while women have an axial distribution of the lesions. If we look at temporomandibular joint (TMJ) involvement, epidemiological data on the involvement of the TMJ are hard to find. Few studies on therapeutic management and the related impact on the quality of life are reported in the literature. Given the morpho-functional peculiarities of the TMJ and the different pain burdens between male and female genders, when manifestation of psoriatic arthritis occurs, clinicians should face it using a multidisciplinary approach for a correct diagnosis and successful treatment. This review aims to examine the diagnostic signs of psoriatic arthritis in the TMJ, the eventual variations of this disease in male and female patients, and the therapeutical strategies. The coordination of different specialties is fundamental to the remission of clinical symptoms and lesion regression.

## 1. Introduction

Psoriatic arthritis (PsA) is a chronic autoimmune condition characterized by joint inflammation and skin involvement. It is a distinct form of arthritis associated with psoriasis, a chronic skin disorder characterized by red, scaly patches (5% to 42%) [1,2]. PsA affects approximately 30% of patients with psoriasis, and its prevalence ranges from 0.1% to 1% in the general population. Both genetic and environmental factors contribute to the development of PsA, and it can occur at any age, although it most commonly manifests between the ages of 30 and 50 [3]. 

The temporomandibular joint (TMJ) is a bilateral joint located in front of the ears, connecting the jawbone (mandible) to the skull. It is a complex joint that allows for various movements, including opening and closing of the mouth, chewing, speaking, and yawning. The TMJ comprises the condyle of the mandible, the articular disc, and the glenoid fossa of the temporal bone. It is surrounded by muscles, ligaments, and a synovial membrane, which provide stability and facilitate smooth movement [4]. The significance of the TMJ in daily activities cannot be overstated. It plays a fundamental role in essential functions such as eating, speaking, and facial expression. Any dysfunction or impairment of the TMJ can have a profound impact on a person’s quality of life. Simple tasks like biting into food or engaging in conversations may become challenging or even painful for individuals experiencing TMJ disorders [5]. 

PsA affecting the TMJ represents a unique subset of PsA manifestations that requires special attention. Indeed, PsA more commonly affects peripheral joints such as the hands, feet, and spine than TMJ. The consequences of TMJ involvement in PsA can be debilitating, significantly affecting an individual’s ability to eat, speak, and perform daily activities. Moreover, the pain and functional limitations associated with TMJ involvement can lead to psychological distress and a reduced overall quality of life. The form involving the joints is present in about 25% of patients with psoriasis and psoriatic arthritis (PsA) [6,7]. 

Understanding the implications of PsA on the TMJ is essential for healthcare providers involved in the diagnosis and management of PsA patients. Indeed, temporomandibular disorders (TMDs) are more frequent in psoriatic patients than in the general population [8]. By recognizing the signs and symptoms of TMJ involvement, clinicians can make accurate diagnoses and develop appropriate treatment plans tailored to the individual’s needs. Furthermore, raising awareness among both medical professionals and patients about the potential impact of PsA on the TMJ can promote early intervention and prevent unnecessary delays in seeking appropriate care.

In this comprehensive review, we aim to explore the various aspects of PsA affecting the TMJ, including the psoriasis pathogenesis, the PsA affecting TMJ clinical presentation, diagnostic approaches, and available treatment strategies. In addition, given the differences in terms of pain receptor distribution and hormonal fluctuations, a peculiar attention is given to the potential gender differences in the manifestation of PsA. 

## 2. Material and Methods

The present review is a narrative and comprehensive review type. In order to provide reliable and trustable information, the review was performed using a search strategy and protocol to select the best items in the literature. 

### 2.1. Search Strategy 

The search strategy relied on the use of the Pubmed, Web of Science, and Scopus databases. The search strategy was conducted using the following search terms: “Psoriasis”, “Psoriatic arthritis”, “Temporomandibular joint”, “Temporomandibular disorders”, “Pathogenesis”, and “Gender differences”. Items available in the English language published by September 2023 were considered. 

### 2.2. Inclusion and Exclusion Criteria 

Clinical studies, epidemiological studies, reviews and systematic reviews, case series, and case reports were included in the present review. Conference proceedings, abstracts in journals and articles published in a non-English language were excluded.

### 2.3. Items Selection

The crucial and more adherent items were independently evaluated by S.B. and D.G. When there was a disagreement between the two authors, a third author, S.M., was consulted. 

In the case of a lack of information or uncertainties, the corresponding authors were contacted and asked for clarification. In the case that this possibility was unavailable, the item has been excluded. 

### 2.4. Extraxted Information Organization 

The selected items were organized in a standard document containing the authors’ names, year of publication, study type, and main provided information and conclusions. 

Therefore, the drafted data were organized in “General Pathogenesis of PsA”, “Pathogenesis of Psoriatic Arthritis Affecting the TMJ”, “Clinical Presentation of Psoriatic Arthritis in the TMJ”, “Diagnosing TMDs associated with PsA”, “Therapies for TMDs Associated with PsA”, “Considerations in management of Psoriatic Arthritis and TMJ Involvement”, and “Psychosocial and Emotional Implications”. 

## 3. General Pathogenesis of PsA 

The pathogenetic mechanisms underlying PsA are still not fully understood; however, studies have revealed an interaction between genetic patterns (HLA loci) and environmental factors, which can trigger the inflammatory pathways leading to arthropathy [8]. The inflammation of the synovial membrane is indeed a typical sign of PsA. 

In particular, angiogenesis due to the inflammatory process is characterized by increased blood vessels in the synovial layer [9]. The pattern of the vessels appears tortuous, dilated, elongated, and immature, which suggests a prolonged activation of the endothelium, ready to respond to the inflammatory status [10]. 

This mechanism increases the metabolism of the synovial tissues, altering their oxygen consumption and making the environment hypoxic, despite the increased vascularization [11].

The low level of oxygen represents an activation signal for the release of inflammatory molecules such as cytokines, changing the adaptation, survival, and activation of T immune cells and macrophages involved in inflammation [12]. 

Hypoxia alters the cellular metabolism of the immune system cells and the synovial cells [13]. Indeed, the synovial cells present altered mitochondria, increased levels of glycolytic enzymes, and damage due to oxidative stress and the activation of fibroblast-like synoviocytes [13,14]. 

Synoviocytes typically proliferate and invade surrounding tissues, and as a consequence, the synovial tissue appears as a tumor-like entity that is potentially able to damage the articular cartilage and bone tissue [15]. 

The proliferation and the invasiveness are nourished by the growth factors and cytokines produced by the inflammatory cells, inducing a vicious circle: hypoxia induces fibroblast-like synoviocytes, which induce angiogenesis, which calls for inflammatory cells, which produce pro-inflammatory cytokines, and which induce the proliferation of fibroblast-like synoviocytes [13]. 

As a morphological result, the cartilage and bone are consumed and subject to erosion: the cartilage goes into fibrillation and the chondrocytes undergo apoptosis, narrowing the joint spaces, which is a pathognomonic sign on X-rays [16]. 

The bone lesions are represented by erosions and disordered proliferation on the periosteal surfaces [16]. The erosions are due to the activation of osteoclasts (by cytokines such as IL-17, IL-22, and IL-23) directed at the articular bone margin [17]. 

In addition, IL-22 is correlated with other bone lesions; IL-22 was found to improve osteoblast functionality by up-regulating the expression of osteogenic factors, including Wnt-3a and BMP4 [18]. 

## 4. Pathogenesis of Psoriatic Arthritis Affecting the TMJ

The pathogenesis of psoriatic arthritis (PsA) affecting the temporomandibular joint (TMJ) involves a complex interplay of immunological factors, genetic predisposition, pro-inflammatory cytokines, and immune dysregulation [19]. Understanding these underlying mechanisms is crucial for developing targeted therapeutic strategies and improving patient outcomes.

PsA is considered an autoimmune disorder as it is characterized by an aberrant immune response targeting the body’s own tissues [20]. In genetically susceptible individuals, exposure to environmental triggers, such as infections or stress, can initiate an immune response that leads to chronic inflammation in various joints, including the TMJ [21].

Genetic studies have identified several susceptibility genes associated with PsA, including HLA-B27, HLA-Cw6, and IL23R [8]. These genes play a role in immune regulation and the activation of inflammatory pathways. HLA-B27, in particular, has been linked to a more severe disease course and increased risk of axial involvement in PsA. However, the genetic factors specifically related to TMJ involvement in PsA remain less understood and require further investigation.

Pro-inflammatory cytokines, such as tumor necrosis factor alpha (TNF-α), interleukin 17 (IL-17), and interleukin 23 (IL-23), play a central role in the pathogenesis of PsA affecting the TMJ [22]. These cytokines are produced by various immune cells, including T cells, macrophages, and dendritic cells, and contribute to the inflammatory cascade observed in PsA. Elevated levels of TNF-α, IL-17, and IL-23 have been found in the synovial fluid and tissues of affected TMJs [23]. 

Immune dysregulation is another critical factor in the pathogenesis of PsA affecting the TMJ. In PsA, there is an imbalance between pro-inflammatory and anti-inflammatory mechanisms, leading to persistent inflammation and tissue damage. Regulatory T cells (Tregs), a subset of immune cells involved in maintaining immune tolerance, play a crucial role in controlling immune responses and preventing excessive inflammation. Reduced Treg function and numbers have been observed in PsA, contributing to the dysregulated immune responses and disease progression. Restoring Treg function may hold therapeutic potential in managing TMJ involvement in PsA [24].

Another important sign related to psoriatic arthritis is bone marrow edema, which is caused by the infiltration of the underlying marrow with inflammatory cells; bone marrow edema is well observed on magnetic resonance imaging [25]. 

The interaction between psoriasis, arthritis, and TMJ involvement further contributes to the pathogenesis of PsA affecting the TMJ. 

Moreover, the presence of psoriasis in individuals with PsA may influence the severity and progression of TMJ involvement. Psoriasis severity, as measured by the Psoriasis Area Severity Index (PASI) [26], has been associated with an increased risk of TMJ arthritis in PsA patients; this suggests that the extent and activity of psoriatic skin disease may correlate with the severity of joints inflammation and its associated symptoms [27].

## 5. Clinical Presentation of Psoriatic Arthritis in the TMJ 

PsA affecting the TMJ presents with a variety of symptoms and dysfunctions that can significantly impact an individual’s daily life [28]. 

One of the hallmark symptoms of TMJ involvement in PsA is pain. Patients may experience pain in and around the TMJ, which can be localized or radiate to the surrounding areas, including the face, temple, and neck [29]. The pain is often described as dull, aching, or throbbing and may be aggravated by jaw movement, chewing, or speaking. It can vary in intensity from mild discomfort to severe, debilitating pain, significantly impacting an individual’s ability to perform routine activities [29].

In addition to pain, joint swelling is commonly observed in PsA affecting the TMJ. The swelling may be visible or palpable, indicating inflammation and synovitis within the joint. The presence of joint swelling can contribute to further discomfort, a limited range of motion, and difficulty in fully opening or closing the mouth. Some individuals may notice clicking, popping, or grating sounds during jaw movement, known as crepitus, which may be indicative of joint damage and degeneration [29].

Limited jaw movement is a significant functional impairment associated with TMJ involvement in PsA. The inflammation and swelling in the TMJ can lead to stiffness and reduced mobility, causing difficulties in opening the mouth wide, chewing, and performing other essential functions. Individuals may experience a sensation of their jaw getting “locked” or “stuck” in a certain position, which may require manual manipulation or self-adjustment to restore normal jaw movement [30].

The impact of PsA affecting the TMJ extends beyond the joint itself and can have profound effects on eating, speaking, and overall quality of life. The pain, limited mobility, and discomfort associated with TMJ involvement can make eating a challenging and painful task. Chewing solid foods, biting into food, and opening the mouth wide enough to take large bites can become particularly difficult. Consequently, individuals may develop dietary restrictions or preferences for softer or easily chewable foods to minimize discomfort [30].

Speaking can also be significantly affected by TMJ involvement in PsA. The pain, joint stiffness, and limited jaw movement can interfere with proper articulation and pronunciation. Some individuals may experience difficulties with enunciating certain sounds or words, leading to speech impediments or a perceived slurring of speech. This can impact communication, social interactions, and self-confidence [30].

### Evaluation of Eventual Gender Differences in TMD-PsA

PsA exhibits gender differences in terms of clinical presentation, disease severity, and underlying factors. The approach to TMD-PsA should consider these differences to optimize the diagnosis, treatment, and management of PsA with TMJ involvement [31].

As regards the prevalence and variation of PsA between males and females, the literature reports contrasting data. Indeed, the gender distribution of PsA is still under debate: Love et al. observed a higher ratio of females/males in an Icelandic population [32]. In contrast, Nossent et al. observed a higher prevalence of males with PsA [33]. However, the growing attention on the influence of gender on the pathogenesis and management of the disease [34] has also induced thorough investigations on gender differences between them in PsA [35,36]. 

The exact reasons for these variations remain unclear and may be influenced by factors such as study design, geographic location, and patient population [34,35,36,37]. Furthermore, this pathology could be underrated due to the different aspecific signs that could affect or be experienced by male or female patients; recent studies showed that the PsA disease phenotype differs between males and females, but further investigation is necessary to fill the gap [35,37]. In particular, several preclinical studies and studies on animal models demonstrate a sexual dimorphism to nociceptive stimuli due to the presence of hormone receptors (estrogen and prolactin) on the dorsal root ganglia, in the fibers innervating joints, and on the central nervous system structures that inhibit and modulate pain perception [38,39].

Gender differences in the clinical presentation of PsA with TMJ involvement have been observed. Males tend to present with more peripheral joint involvement, such as inflammation of the larger joints, including the knees, ankles, and wrists. On the other hand, females often exhibit a higher prevalence of axial involvement, which includes the spine, sacroiliac joints, and TMJ [40]. The reasons for this variation in joint involvement patterns between genders are not well understood and may be influenced by both hormonal and genetic factors. Disease severity in PsA may also exhibit gender differences. Some studies suggest that males with PsA may experience more severe joint inflammation and damage, leading to increased disability and functional impairment. Females, on the other hand, may be more likely to report higher levels of pain, fatigue, and psychological distress associated with PsA [40]. However, these findings are not consistent across all studies, and further research is needed to fully elucidate the gender-specific differences in disease severity.

Hormonal factors play a role in the gender disparities observed in PsA with TMJ involvement. It has been hypothesized that the hormonal fluctuations experienced by females, particularly during reproductive years and menopause, may contribute to the differences in disease presentation and severity. Estrogen, a hormone with immunomodulatory effects, has been suggested to influence the inflammatory response and joint damage in PsA. Fluctuations in estrogen levels during the menstrual cycle and pregnancy may impact disease activity and symptoms. However, the exact mechanisms through which hormones influence PsA with TMJ involvement require further investigation [30].

Genetic factors also contribute to gender disparities in PsA. Studies have identified specific genetic variants associated with PsA susceptibility, and some of these genetic loci may exhibit gender-specific effects. For instance, mutation in the HLA-B27 gene, which is strongly associated with axial involvement in PsA, has been reported to have a higher prevalence in males [8]. Other genetic factors that modulate immune responses and inflammation may also contribute to gender differences in disease manifestation and severity. However, the interplay between genetics, hormones, and the immune system in PsA with TMJ involvement is complex and requires more research to fully understand.

In addition to hormonal and genetic factors, sociocultural and behavioral factors may also contribute to gender differences in PsA with TMJ involvement. Variations in healthcare-seeking behaviors, access to health care, and adherence to treatment regimens may influence disease outcomes and management. 

There are several forms of psoriasis: type I, type II, and psoriatic arthritis (PsA). Among these three forms, the latter has an increased disease burden [41] in females. 

The increasing attention to medical history and rheumatological disease is increasing the number of diagnosed cases of PsA-TMD. 

Women affected by PsA present with multiple articular lesions, a significant loss of functionality, and increased fatigue in their daily movements [8,36,41]. 

The female sex is generally more exposed to developing TMD, and the disorder harms the patient’s quality of life. A recent review from Stinson et al. showed how the sex steroid hormones such as estrogen affect the condylar morphology and health [42]. In addition, the Hauru et al. study, employing Fourier-transform infrared imaging, showed how low levels of estradiol affected the proteoglycal levels in the cartilage of the mandibular condyle [43]. 

TMDs influence patients’ quality of life, specifically in female patients who may have a higher perception of pain; in this regard, in their study, Christidis et al. reported the higher expression of the serotonin receptor in masseter muscles in women, which explains the increased sensitivity of females to pain, especially in the masseter region [44]. 

In addition, females present with a peculiar sensitivity to pain stimulation because of sex hormonal peaks and changes [45], making TMJ arthropathy a high-burden disease. 

Indeed, as reported by Quinelato et al., estrogen not only acts on bone cells and tissue physiology but also on the pain and serotonin receptors [46]. Given the role played by the pain and the inflammatory processes, hormonal changes expose the female gender to a higher susceptibility and pain development than the male gender [47]. 

In addition, the age of diagnosis is different between males and females. Female patients usually present at one of two critical peaks of age: one usually overlaps with the peak of the reproductive age [37], and another one is the post-menopausal age range, indicating an association with hormonal fluctuations [37]. If the reproductive and the menopausal periods represent physiological changes in hormonal concentrations, specific therapies, such as ovarian hyperstimulation in reproductive technique (ART) protocols, which artificially change the hormonal fluctuations and peaks, can significantly impact the course and onset of psoriasis [39]. 

In the study conducted by Wilson et al., the mean age was 43.3 ± 17.1 years and 50% of the related population were women [48].

Finally, as Gottlieb A. [49] showed, PsA has a higher negative impact on women than men regarding emotional and psychological well-being. 

## 6. Diagnosing TMDs Associated with PsA 

There are literature reports of TMD associated with PsA, but there are few coherent statistical studies on its frequency [8,50,51,52,53,54], making the PsA forms affecting the TMJ rare. The TMJ is reported as a possible anatomical site of chronic pain with a high impact in moderate to severe pain cases and consequent disability [1,50,51,52,53,54].

The clinical diagnosis of PsA-TMD is based on medical history assessment, particularly in terms of rheumatological disease and physical examination of the TMJ and the masticatory muscles. The final diagnosis is completed with radiological evidence [55]. Thus, the diagnostic approach includes the following scheme: medical history, with a focus on patients affected by psoriasis, and physical examination of the TMJ. The clinical signs are arthralgia, crepitus, and rarely, ankylosis on both sides [55]. The aspecific symptoms include pain, functional limitations, and muscular effects [48,51,56,57], with general orofacial pain, headache, and myalgia at the masticatory muscles and sleep disorders [58,59,60].

Radiological findings that are consistent with the diagnosis include cortical erosion, the narrowing of the articular space, the flattening of the articular surfaces, and osteophytic lesions [55].

The imaging methods capable of showing the pathological signs in detail are computed tomography to assess the degree of damage to the bones and magnetic resonance imaging to highlight the health of the soft tissues [61]. As reported by Wang et al., the most commonly occurring lesions found by radiology imaging are bone erosions [62].

Unfortunately, the diagnosis is tricky due to the clinical presentation of PsA; other similar pathologies are degenerative osteoarthritis and rheumatoid arthritis. However, according to Wang et al. [62], some radiological details can allow us to differentiate PsA from other inflammatory diseases; for example, in patients affected by osteoarthritis, the erosions occur in the central portion of the condyles, while in PsA patients, the erosive lesions can occur at the central or margin parts of the articular surfaces. In addition, proliferative bone lesions, such as enthesitis, are typical signs of PsA [62].

Diagnosis, when the medical history is negative for PsA, is complemented by genetic and rheumatoid factor analyses and by the characteristic course of the disease, since the degeneration due to rheumatoid arthritis is usually faster than PsA [56]. 

## 7. Therapies for TMDs Associated with PsA 

The therapy for TMDs associated with PsA is aimed at controlling the disease, which can be considered a manifestation of a systemic disease. Therefore, the first-line treatment is pharmacological [53]. Rheumatology specialists should be informed of the diagnosis to discuss the right pharmacological therapy with the patient to control the disease [57]. 

As previously reported [53], the first pharmacological plan usually includes the administration of non-steroidal anti-inflammatory drugs (NSAIDs) and steroids. If the degree of the PsA is considered moderate–severe, the pharmacological therapy can include the simultaneous administration of methotrexate, which is much more tolerated for long-term usage. If this is the case, intraarticular pharmacological administration can be used [63].

Biological therapy represents the second line of pharmacological therapy, usually indicated in patients with moderate–severe PsA, who cannot be given NSAIDs or methotrexate [63]. These biological therapies include anti-tumor necrosis factor (anti-TNF) agents (infliximab, etanercept, adalimumab, and golimumab), interleukin (IL)-12/IL-23 inhibitors (ustekinumab), and IL- 7 inhibitors (brodalumab) [49].

TNF-alpha is a pro-inflammatory cytokine that was first identified in the etiopathogenesis of autoimmune and inflammatory diseases, including psoriasis and PsA [13]. This cytokine, produced during the inflammation process, was found to be responsible for the damage to osteoblasts and chondrocytes [51]. Biological therapy targeting TNF-alpha has shown promising results; however, studies reported side effects due to the biological therapy, such as cancer and osteonecrosis of the jaw, which, in the case of TMD, could worsen oral functionality [63]. 

In cases of PsA-TMD, in addition to the available pharmacological therapies first aimed at reducing the pain, co-adjuvant therapies, such as gnathological and physical therapies, may help reduce pain and recover functionality [49,56]. 

In addition, occlusal therapy aims to equilibrate and relieve the occlusal load on the oral musculoskeletal system, improve TMJ functionality, and prevent any further lesions to the TMJ [64]. 

Another therapy option for treating PsA-TMD is surgery, which is reserved for severe cases where pharmacological therapy was not successful [53]. The surgical therapy options include arthrocentesis, arthroplasty, condylotomy, and custom-made, total TMJ replacement [65, 66, 67]. 

### 7.1. Differences in Therapies between TMDs Associated or Not with PsA 

TMDs are a heterogeneous group of disorders that affect the temporo-mandibular joint, involving the muscular function and, thus, the phases of mastication and phonation. The main therapy for TMDs is represented by gnathological therapy [67]: considering that the main symptom of the TMDs is the pain related to the pathological function of the masticatory muscles and the temporo-mandibular joint, the first choice in therapy is the conservative treatment, which includes different techniques like massage therapy, individually fabricated occlusal splints, manual therapy and taping, warming/cooling of aching joints, and light and laser therapy. In the case of a severe grade of TMDs, the treatment also includes drugs or surgical therapy aimed at restoring the joint [68].

In the case of TMDs associated with PsA and the presence of an inflammatory pathological mechanism affecting patients due to the rheumatic disease, the pharmacological therapy is fundamental [53], and it could be associated with a conservative therapy.

### 7.2. Arthrocentesis

Arthrocentesis is a minimally invasive surgical procedure that involves the insertion of needles into the affected TMJ to irrigate the joint space and remove inflammatory mediators and debris. This procedure aims to alleviate pain and improve joint function by reducing inflammation and restoring the balance within the joint [65]. Arthrocentesis has shown promising results in patients with PsA-TMD, particularly in cases where conservative treatments have failed to provide adequate relief [66].

### 7.3. Arthroplasty

Arthroplasty, also known as joint reconstruction or joint replacement, is a surgical option for severe cases of PsA-TMD that involve significant joint damage. During arthroplasty, the damaged or diseased components of the TMJ are removed and replaced with artificial prostheses to restore joint function and alleviate pain [65,66]. This procedure is typically reserved for patients with advanced joint deterioration and can provide substantial improvements in joint mobility and overall quality of life.

### 7.4. Condylotomy

Condylotomy is a surgical technique used to address TMJ disorders by modifying the shape or position of the mandibular condyle. This procedure aims to correct functional abnormalities and improve jaw alignment, thereby reducing pain and restoring normal jaw function [65,66]. Condylotomy can be performed using various approaches, including open or closed techniques, depending on the specific needs of the patient. It is often considered in cases where other surgical options may not be suitable or necessary.

### 7.5. Custom-Made, Total TMJ Replacement

In cases of severe joint damage and dysfunction that cannot be effectively treated by other surgical interventions, custom-made, total TMJ replacement may be considered. This procedure involves the complete removal of the diseased joint components and the placement of a custom-designed prosthetic joint. The prosthetic joint is tailored to fit the patient’s anatomy and aims to restore normal joint function, alleviate pain, and improve overall jaw mobility [65,66]. Custom-made, total TMJ replacement is a complex surgical procedure that requires careful patient selection and extensive preoperative planning.

It is important to note that the choice of surgical intervention for PsA-TMD depends on the individual patient’s condition, severity of symptoms, and response to conservative treatments. A thorough evaluation by a multidisciplinary team, including rheumatologists, oral, and maxillofacial surgeons and other relevant specialists, is essential to determine the most appropriate treatment approach for each patient.

## 8. Considerations in Management of Psoriatic Arthritis and TMJ Involvement

When managing patients whose TMJ is involved in PsA, gender has to be considered, also due to the possibility of pregnancy and hormonal changes in female patients. 

Pregnancy itself can have variable effects on the course of PsA, with some women experiencing improvement in symptoms during pregnancy, while others may experience disease flares. Hormonal changes during pregnancy, particularly fluctuations in estrogen levels, can influence the immune system and potentially impact TMJ symptoms [69]. It is important for healthcare providers to closely monitor TMJ symptoms during pregnancy and adjust the treatment plan accordingly.

The safety of medications used to manage PsA with TMJ involvement during pregnancy and breastfeeding is a critical consideration. Many traditional disease-modifying antirheumatic drugs (DMARDs) and biologic agents have limited safety data in pregnant or breastfeeding women. Rheumatologists, in collaboration with obstetricians and gynecologists, must carefully evaluate the potential risks and benefits of continuing or modifying medication regimens during pregnancy and breastfeeding. In some cases, treatment modifications may be necessary to ensure the well-being of both the mother and the baby [70].

### Multidisciplinary Care Involving Rheumatologists, Gynecologists, and Dentists

Managing PsA with TMJ involvement in females requires a multidisciplinary approach. Rheumatologists, gynecologists, and dentists play essential roles in coordinating care, addressing hormonal influences, managing pregnancy-related concerns, and optimizing TMJ function. Collaborative communication among healthcare providers ensures comprehensive and individualized care throughout pregnancy, postpartum, and beyond.

## 9. Psychosocial and Emotional Implications

PsA with TMJ involvement can have significant psychosocial and emotional implications for females. Living with chronic pain, functional limitations, and the impact on daily activities can contribute to emotional distress, anxiety, and depression. It is important for healthcare providers to address these psychosocial aspects of the disease and provide the appropriate support [71].

Understanding the impact of chronic pain on mental health and emotional well-being is crucial [72]. Females with PsA with TMJ involvement may experience challenges in various aspects of their lives, including personal relationships, professional pursuits, and overall quality of life. Healthcare providers should assess and monitor mental health status, provide counseling, and refer patients to mental health professionals when needed.

Developing effective coping strategies and establishing support systems are vital for females with PsA with TMJ involvement. Encouraging patients to engage in activities that promote relaxation, stress reduction, and overall well-being can be beneficial. Support groups, patient education programs, and online communities can provide a sense of belonging, empathy, and shared experiences, which can positively impact emotional well-being [73,74].

Holistic care and patient education are paramount in managing PsA with TMJ involvement in females. Providing comprehensive education about the disease, treatment options, self-management strategies, and available resources empowers patients to actively participate in their own care. Promoting a holistic approach that considers physical, mental, and emotional aspects of health can lead to better overall outcomes and improved quality of life.

## 10. Future Research Directions

Further research is necessary to better understand the specific needs and optimal management strategies for females with PsA with TMJ involvement. Identifying novel biomarkers that can aid in early detection, predict disease progression, and guide treatment decisions is an important area of investigation. Gender-specific biomarkers may provide insights into the distinct pathogenic mechanisms, disease patterns, and treatment responses observed in females.

Developing gender-specific treatment guidelines for PsA with TMJ involvement is another crucial area for future research. Tailoring treatment strategies to address the unique hormonal, genetic, and clinical characteristics of females can help optimize outcomes. Conducting large-scale clinical trials and longitudinal studies with a focus on female participants is essential to generate robust evidence and inform evidence-based guidelines.

Furthermore, research should explore the impact of hormonal interventions, such as hormone replacement therapy, on TMJ symptoms and disease progression in females with PsA. Investigating the interplay between hormonal fluctuations, inflammation, and joint damage can provide valuable insights into disease mechanisms and potential therapeutic approaches.

## 11. Conclusions

In conclusion, given the crucial role of TMJ in the functionality of the stomatognathic system, it is possible to speculate on the presence of a rheumatological disease affecting it, which might definitely affect the quality of life of patients involved. 

The few literature examples available pose unique challenges regarding the treatment and the improvement of the clinical signs that could affect the function of the temporomandibular joint and, thus, the phases of chewing and phonation. Recognizing the clinical presentation, understanding the underlying pathogenesis, and tailoring treatment strategies are essential in managing this condition effectively. Additionally, it is necessary to conduct more studies, considering the emerging gender differences, clinical manifestations, and treatment responses, in order to optimize care for both males and females with PsA affecting the TMJ. By fostering interdisciplinary collaboration and investing in further research, healthcare providers can pave the way for improved diagnosis and treatment and, ultimately, enhance patient outcomes.

## Data Availability

Data will be available from Sara Bernardi upon kind request.

## Abbreviations

PsA: psoriatic arthritis, TMJ: temporomandibular joint, TMD: temporomandibular disorder, NSAID: non-steroidal anti-inflammatory drug.
